# Comparison of mortality and hospitalisation rates amongst older adults residing in professional foster families versus nursing homes: two parallel observational studies

**DOI:** 10.1093/ageing/afaf304

**Published:** 2025-10-18

**Authors:** Denis Boucaud-Maitre, Nadine Simo-Tabué, Océane Pic, Laurys Letchimy, Roxane Villeneuve, Christine Rambhojan, Larissa Vainqueur, Leila Rinaldo, Moustapha Dramé, Jean-François Dartigues, Helene Amieva, Maturin Tabué-Teguo

**Affiliations:** Centre Hospitalier Le Vinatier, DRCI, Bron, France; Université des Antilles, EPICLIV, Fort-de-France, Martinique; Université des Antilles, EPICLIV, Fort-de-France, Martinique; CHU de Martinique, Geriatric Unit, Fort-de-France, Martinique; Université de Bordeaux, Inserm U1219 Bordeaux Population Health Center, Talence, France; Université des Antilles, EPICLIV, Fort-de-France, Martinique; CHU de Martinique, Geriatric Unit, Fort-de-France, Martinique; CHU Guadeloupe, Geriatric Unit, Pointe-a-Pitre, Guadeloupe; CHU Guadeloupe, Geriatric Unit, Pointe-a-Pitre, Guadeloupe; CHU Guadeloupe, Geriatric Unit, Pointe-a-Pitre, Guadeloupe; CHU Guadeloupe, Geriatric Unit, Pointe-a-Pitre, Guadeloupe; Université des Antilles, EPICLIV, Fort-de-France, Martinique; CHU de Martinique, Geriatric Unit, Fort-de-France, Martinique; Université de Bordeaux, Inserm U1219 Bordeaux Population Health Center, Talence, France; Université de Bordeaux, Inserm U1219 Bordeaux Population Health Center, Talence, France; Université des Antilles, EPICLIV, Fort-de-France, Martinique; CHU de Martinique, Geriatric Unit, Fort-de-France, Martinique

**Keywords:** nursing homes, foster families, mortality, hospitalisation, cognition, dependency, older people

## Abstract

**Background:**

There is an urgent need to offer alternatives to nursing homes. Amongst them, professional foster families with paramedical supervision have never been studied.

**Objectives:**

This study aimed to compare the 1-year mortality, hospitalisation and emergency department visit rates between older adults in foster families and those in nursing homes.

**Design:**

Two parallel cohort studies conducted in foster families (*n* = 107) and nursing homes (*n* = 332).

**Methods:**

Adults aged ≥60 years were followed for 1 year, with assessments at baseline, 6 and 12 months. Primary outcomes included 12-month mortality, hospitalisations and emergency visits without hospitalisation. Secondary outcomes included cognitive decline [Mini-Mental State Examination (MMSE)], functional dependence [activities of daily living (ADL)], and health-related quality of life (EQ-5D).

**Results:**

Baseline characteristics, including comorbidities and cognitive impairment (MMSE ≤18), were similar across groups. However, full functional dependence (ADL = 0) was more frequent amongst foster family residents (35.4% versus 18.2%; *P* = .01). After 12 months, mortality was 24.2% in foster families and 21.9% in nursing homes [adjusted odd ratio (OR) = 0.83; 95% CI, 0.42–1.66; *P* = 0.599]. Hospitalisation occurred in 6.1% versus 8.6% (adjusted OR = 0.74; 95% CI, 0.28–2.00; *P* = .547), and emergency visits in 9.1% versus 9.3% (adjusted OR = 0.94; 95% CI, 0.37–2.37, *P* = 0.889). Cognitive decline was similar. Foster family residents showed greater functional decline (β = −0.69; *P* < .001) but better quality of life maintenance (β = 0.08; *P* = .004).

**Conclusion:**

Professional foster families are a viable alternative to nursing homes, achieving comparable health outcomes at 1 year and at lower cost. Their development should consider cultural, clinical, and socioeconomic contexts.

## Key Points

- Professional foster families for dependent older adults perform at least as efficiently as nursing homes.
- This model requires a partnership-driven organisation of services to ensure effective coordination.
- It may play a pivotal role in shaping the healthcare systems of many countries.

## Introduction

The greatest global demographic challenge in the coming decades is not population growth but ageing. While the vast majority of older adults wish to and will be able to age at home, this is unfortunately not always possible. Public policies in many countries are geared toward home ageing with associated care, known as ageing in place [1]. However, significant disparities in access to these services persist, often due to economic or geographic barriers [2]. At the stage of frailty, which could be associated with social or familial isolation (such as the loss of a spouse), alternatives exist—such as senior housing—or are being tested, including small-scale homelike facilities [3]. However, when older adults transition into dependency, particularly with mild to severe cognitive impairments, few options are available outside of nursing homes. In high-income countries, hospital-at-home [4], telemedicine [5] or care coordination [6] are being implemented. In low- and middle-income countries, which are expected to account for 80% of the world’s older adult population by 2050 [7], the nursing home model has been developed to a very limited extent. The availability, cost, quality and role of these facilities vary significantly across regions, depending on local resources and cultural contexts. As a result, older adults in these countries are primarily cared for by their families due to a lack of alternatives [8]. With increasing urbanization and the migration of younger generations—who often serve as family caregivers—an increasing number of older adults will live alone, further driving the need for residential care solutions beyond home-based care when dependency arises.

The World Health Organisation underscores the importance of integrating care services primarily through community-based and primary care frameworks to support ageing individuals within their residential environments [9]. In light of the significant global demand for long-term care facilities for dependent older adults, further highlighted by excess mortality during the pandemic, it is imperative to investigate alternatives to traditional nursing homes. This exploration aims to diversify care options according to the preferences of older adults and to develop models tailored for countries that do not wish to adopt the nursing home paradigm. In this context, foster families for dependent older adults may represent a promising yet underexplored strategy [10]. In the French West Indies (Caribbean islands), professional foster families for older adults have existed for over 30 years, driven by local health policies and the social, cultural and anthropological specificities of the region. This model also emerged from the limited availability and high cost of nursing homes, as well as the strong cultural aversion to institutionalised care.

Each foster family accommodates one to three older adults, providing them with a private room, meals and daily activities. Medical follow-up is frequently integrated, with nurses visiting once or twice a day, physiotherapists providing regular care and a general practitioner ensuring ongoing supervision. Additionally, this model is characterised by its structured organisation under governmental oversight, including an initial 60-hour training programme and official accreditation for caregivers. The authorities directly compensate foster caregivers with a fixed monthly allowance of €1500 per resident [11] while the mean cost for a nursing home resident is about €3000 in the same area.

This innovative care model could play a significant role in healthcare systems across various countries if robust data were to demonstrate its effectiveness. Although foster family programmes exist in many countries, the available studies on their outcomes are outdated. A Cochrane meta-analysis published in 2015 [12] identified only three studies [13–16] conducted nearly 40 years ago, comparing foster family care with nursing home or home-based care. The authors concluded that the current literature is insufficient to assess the effectiveness of foster families due to small sample sizes, significant methodological biases and heterogeneous baseline characteristics of residents.

In this context, we initiated the KASA project, which aimed to conduct two parallel longitudinal studies to assess the health trajectories of older adults living either in foster families (KASAF study, i.e. KArukera study of Ageing in Foster families) or in nursing homes (KASEHPAD study, i.e. KArukera study of ageing in EHPAD). The inclusion data from these studies indicated that the residents’ profiles were similar in terms of age, sex, cognitive impairment, nutritional status and neuropsychiatric disorders [11, 16]. Moreover, comparative results suggested that at baseline, health-related quality of life (HrQoL) was not associated with living in a nursing home or a foster family. However, subjective quality of life was 19.5 points lower in nursing homes compared to foster families [17]. In this article, we present the 1-year follow-up data from these two studies.

The primary objective was to compare 1-year mortality, hospitalisation rates and emergency department visits between older adults living in foster families and those residing in nursing homes. The secondary objective was to compare the evolution of cognitive function, disability and HrQoL between the two care models.

## Methods

The KASAF and KASEHPAD studies are twin prospective cohort studies employing identical methods and outcomes to describe the health trajectories of older adults living in foster families (KASAF) and nursing homes (KASEHPAD) over 1 year. The protocol for KASAF [18] and the inclusion data for these two studies [11, 16] have been previously published. To be eligible, participants had to be aged 60 years or older, residing in a foster family in Guadeloupe or a nursing home in the French West Indies (Guadeloupe and Martinique), and covered by French social security. Recruitment for KASAF began in September 2020 and ended in May 2021, while recruitment for KASEHPAD spanned from September 2020 to November 2022. Both older adults and caregivers were interviewed on-site by a geriatrician or clinical research nurse at three time points: (i) at baseline, (ii) at 6 months, and (iii) at 12 months. For older adults, we collected anthropometric measures, cognitive status, depressive and anxiety symptoms, functional abilities, physical frailty, general health status, quality of life and care pathways (hospitalisation, mortality, medical and paramedical consultations). A phone update of the vital status (alive or deceased) and care pathways was carried out at 3 and 9 months after the baseline examination. The KASAF study is registered on ClinicalTrials.gov (NCT04545775). The KASAF study received approval from the French Sud Méditerranée III Ethics Committee on 1 July 2020. The EST 1 French Ethics Committee approved the KASEHPAD study on 2 June 2020. The study was registered on ClinicalTrials.gov on 13 October 2020 (NCT04587466). All the participants or their legal guardians gave informed consent, and all methods were performed in accordance with the relevant guidelines and regulations.

### Data

The following characteristics were extracted from the KASAF and KASEHPAD studies: age, gender, marital status, comorbidities, scores on clinical scales including the Mini-Mental State Examination (MMSE) [19], activities of daily living (ADL) Katz’s scale [20], and HrQoL. The MMSE [21] assesses cognitive function on a scale from 0 to 30. Katz’s scale [22] comprises six items assessing independence in basic ADL: bathing, toileting, eating, locomotion, dressing and incontinence. HrQoL was assessed using the proxy EQ-5D-3L questionnaire [21, 22] reported by the professional caregiver, which includes five dimensions (mobility, self-care, usual activities, pain/discomfort and anxiety/depression) and three response levels (‘no problems’, ‘some problems’ and ‘extreme problems’). In a previous study [17], we found a strong concordance between HrQoL as reported by the older adult and their professional caregiver (correlation coefficient: 0.644). An individual health profile is assigned, and a summary index score is calculated based on societal preference weights for the health state [18]. The French value set for the EQ-5D-3L was employed to compute the EQ-5D Index value [23], with values below zero indicating worse than death and one indicating full health. At each time point (6 months and 12 months), vital status, hospitalisation and emergency admission data were extracted.

### Outcome measures

The primary outcomes were mortality rate, hospitalisation rate and emergency admission rate (not followed by hospitalisation) at 12 months. The secondary endpoint was the evolution of cognition, dependence and HrQoL.

### Supplementary analysis

Due to the potential impact of the COVID-19 crisis on the results, a supplementary analysis was conducted to compare hospitalisation and mortality rates between older adults in nursing homes recruited during the COVID-19 period (2020–2021) and those recruited afterward (2022).

### Patient and public involvement

No older adults or members of the public were involved in the design, analysis or reporting of this study.

### Statistical analysis

Quantitative variables were presented as mean ± standard deviation, median and interquartile range (IQR). Qualitative variables were expressed as percentages. Demographic and clinical characteristics were compared between participants in foster families and nursing homes using independent t-tests for continuous variables and chi-squared or Fisher’s exact tests for categorical variables. Logistic models were used to compare mortality rate, hospitalisation rate and emergency admission rate between both groups, without adjustment (model 1), adjusted for age, gender and ADL score at baseline (model 2), and adjusted for age, gender, ADL score, hypertension and MMSE score at baseline (model 3). Linear mixed regression models were used to compare the evolution of secondary outcome variables (MMSE score, ADL score, and EQ-5D score). The level of significance was set to *P* < .05. R software (R Development Core Team, Vienna, Austria) was used for the data analysis.

## Results

**Figure 1:**
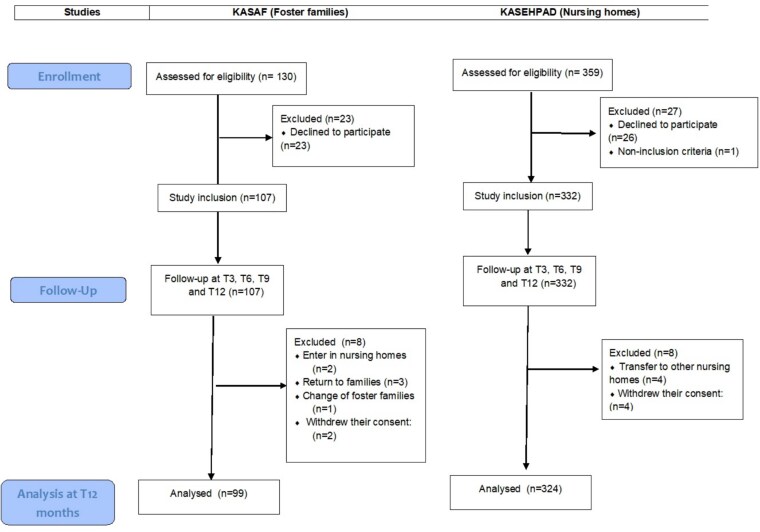
Flowchart of KASAF and KASEHPAD studies.

In total (*n* = 439), 107 older adults were included in the KASAF study run in foster families, and 332 were included in the KASEHPAD study in nursing homes. The participation rates were 82.2% for KASAF and 92.5% for KASEHPAD. During the follow-up of the KASAF study, eight older adults exited the study: two were transferred to nursing homes, three were taken care of by their families, one was transferred to another foster family, and two withdrew their consent. Regarding KASEHPAD, eight older adults exited the study: four were transferred to a nursing home or other care units, and four withdrew their consent (Figure 1).

At inclusion, older adults in foster families had a mean age of 82.0 ± 11.1 years, and those in nursing homes had a mean age of 81.1 ± 10.0 years (*P* = .447). There were more men in nursing homes than in foster families (51.5% versus 39.4%, *P* = .045). No significant difference was found in terms of comorbidities, except for hypertension, which was more frequent in nursing homes (67.9% versus 47.3%; *P* < .001). The prevalence of major cognitive impairment (MMSE score ≤ 18) was statistically similar between the two groups (72.0% versus 75.3%; *P* = .655). Finally, residents were more often severely dependent (ADL score = 0) in foster families (35.4% versus 18.2%, *P* = .001) at inclusion (Table 1).

**Table 1:** Clinical characteristics of older adults living in foster families or nursing homes.

	Total		Older adults in foster families		Older adults in nursing homes		
Characteristics	*n*	Mean or % Median; IQR	n	Mean or % Median; IQR	n	Mean or % Median; IQR	*P*-value
Age (years)	423	81.3 ± 10.3 82.0; 16.0	99	82.0 ± 11.1 85.0; 17.0	324	81.1 ± 10.0 81.0; 15.0	.447
Gender (=men)	423	206 (48.7%)	99	39 (39.4%)	324	167 (51.5%)	.045
*Marital status* —widowed —in a relationship —single or divorced	413	85 (20.6%) 39 (9.4%) 289 (70.0%)	98	20 (20.4%) 12 (12.2%) 66 (67.3%)	315	65 (20.6%) 27 (8.6%) 223 (70.8%)	.550
Education (=low level)	347	298 (85.9%)	89	77 (86.5%)	258	221 (85.7%)	.981
*Length of stay (years)*	400	4.5 ± 4.3 3.3; 3.8	99	4.8 ± 4.9 3.1; 5.3	301	4.4 ± 4.1 3.3; 3.4	.397
Diabetes (=yes)	409	114 (27.9%)	94	23 (24.5%)	315	91 (28.9%)	.479
Hypertension (=yes)	411	260 (63.3%)	93	44 (47.3%)	318	216 (67.9%)	<.001
Ischemic heart disease (ischemic cardiopathy, acute myocardial infarction) (=yes)	405	22 (5.4%)	93	2 (2.2%)	312	20 (6.4%)	.184
Other heart diseases (atrial fibrillation, heart failure, valvular heart disease) (=yes)	402	58 (14.4%)	93	14 (15.1%)	309	44 (14.2%)	.978
Cerebrovascular disease (ischemic or haemorrhagic stroke) (=yes)	407	88 (21.6%)	93	23 (24.7%)	314	65 (20.7%)	.493
Dementia (=yes)	370	218 (58.9%)	83	49 (59.0%)	287	169 (58.9%)	.999
Depression (=yes)	412	94 (22.8%)	95	28 (29.5%)	317	66 (20.8%)	.104
MMSE Mean ± SD Median; IQR MMSE ≤18	346	10.8 ± 9.9 11.0; 19.8 252 (72.8%)	85	8.3 ± 10.0 0.0; 18.0 64 (75.3%)	261	11.7 ± 9.7 12.0; 20.0 188 (72.0%)	.006 .655
ADL Mean ± SD Median; IQR ADL score = 0	417	2.2 ± 2.1 1.5; 3.5 93 (22.3%)	99	1.4 ± 1.7 0.5; 2.2 35 (35.4%)	318	2.4 ± 2.1 2.0; 4.0 58 (18.2%)	<.001 .001
EQ5D index Mean ± SD Median; IQR	406	0.2 ± 0.4 0.2; 0.7	98	0.2 ± 0.4 0.1; 0.7	308	0.3 ± 0.4 0.3; 0.6	.031

### Primary outcomes

Twenty-four (24.2%) deaths occurred in foster families, compared to 71 (21.9%) in nursing homes (unadjusted OR = 1.14, 95% CI: [0.67–1.94], *P* = .629). Adjusted for age, gender and baseline ADL score (model 2, *n* = 417), the OR was 0.85 (95% CI: [0.47–1.51], *P* = .573), and adjusted for age, gender, baseline ADL, hypertension and MMSE scores (model 3, *n* = 331), the OR was 0.83 (95% CI: [0.42–1.66], *P* = .599).

The proportion of hospitalisations was 6.1% (six residents) in foster families and 8.6% (28 residents) in nursing homes (unadjusted OR = 0.68; 95% CI: [0.27–2.70]; *P* = 0.395). Adjusted for age, gender, and baseline ADL score (*n* = 417), the OR was 0.67 (95% CI: [0.26–1.70], *P* = 0.381), and adjusted for age, gender, baseline ADL, hypertension and MMSE scores (*n* = 341), the OR was 0.74 (95% CI: [0.28–2.00], *P* = .547).

The emergency admission rate was 9.1% (nine residents) in foster families and 9.3% (30 residents) in nursing homes (unadjusted OR = 0.98, 95% CI: [0.45–2.14], *P* = .960). Adjusted for age, gender and baseline ADL score (*n* = 417), the OR was 1.11 (95% CI: [0.49–2.49], *P* = .810), and adjusted for age, gender, baseline ADL, hypertension and MMSE scores (*n* = 331), the OR was 0.94 (95% CI: [0.37–2.37], *P* = .889).

For a composite criterion including mortality and hospitalisations, the adjusted OR (model 3) was 0.68 (95% CI: [0.36–1.27], *P* = .219 (Table 2).

**Table 2:** Comparison of deaths, hospitalisations and emergency admission between older adults living in foster families and those in nursing homes at 1 year.

Clinical outcomes	Foster families	Nursing homes	Model 1 (non-adjusted) (*n* = 423)		Model 2 (adjusted on age, gender and ADL score) (*n* = 417)		Model 3 (adjusted on age, gender, hypertension, ADL and MMSE score) (n = 331)	
	99	324	OR (CI 95%)	*P*	OR (CI 95%)	*P*	OR (CI 95%)	*P*
Death	24 (24.2%)	71 (21.9%)	1.14 (0.67–1.94)	.629	0.85 (0.47–1.51)	.573	0.83 (0.42–1.66)	.599
Hospitalisations	6 (6.1%)	28 (8.6%)	0.68 (0.27–1.70)	.395	0.67 (0.26–1.70)	.381	0.74 (0.28–2.00)	.547
Composite endpoint death and hospitalisation	27 (27.3%)	96 (29.6%)	0.89 (0.54–1.47)	.650	0.69 (0.40–1.17)	.163	0.68 (0.36–1.27)	.219
Emergency admissions without hospitalisation	9 (9.1%)	30 (9.3%)	0.98 (0.45–2.14)	.960	1.11 (0.49–2.49)	.810	0.94 (0.37–2.37)	.889

### Secondary outcomes

Regarding cognition, no significant difference was observed in the evolution of the MMSE score between the two groups (adjusted β: −0.70; *P* = .485), nor in the evolution of the percentage of older adults with major cognitive impairment (MMSE ≤18). In terms of dependence, a more significant decrease in the ADL score was observed in foster families (adjusted β: −0.69; *P* < .001), although there was no difference in the evolution of the percentage of older adults in total dependence (ADL score = 0). Finally, the HrQoL score was higher in foster families (β: 0.08; *P* = .004) (Table 3).

**Table 3:** Comparison of cognition, dependency and HrQoL between older adults living in foster families and those in nursing homes at 1 year.

Clinical outcomes	Model 1 (non-adjusted)			Model 2 (adjusted on age and gender)			Model 3^a^ (full adjusted)		
	*n*	β (SE)	*P*	*n*	β (SE)	*P*	*n*	β (SE)	*P*
MMSE Ref: nursing homes	346			346			331		
Score ≤ 18 (Yes/No)		0.33 (1.05)	.755		0.47 (1.09)	.664		−2.50 (1.53)	.103
Continuous score		−3.37 (1.21)	.006		−3.38 (1.20)	.005		−0.70 (1.00)	.485
ADL Ref: nursing homes	417			417			331		
Score = 0 (Yes/No)		0.98 (0.82)	.232		1.05 (0.86)	.225		0.66 (1.29)	.612
Continuous score		−0.90 (0.22)	<.001		−0.82 (0.21)	<.001		−0.69 (0.18)	<.001
EQ5D Ref: nursing homes	406			406			322		
Continuous score		−0.11 (0.05)	.019		−0.09 (0.04)	.044		0.08 (0.03)	.004

For ADL scale, model adjusted on age, gender, hypertension and baseline MMSE score.

For the EQ5D scale, model adjusted on age, gender, hypertension, baseline MMSE and ADL scores.

^a^For MMSE scale, model adjusted on age, gender, hypertension and baseline ADL score.

### Supplementary analysis: impact of COVID crisis on outcomes

No significant difference was found in mortality rate and hospitalisation rate between residents in nursing homes included during the COVID crisis (reference) and those included after the COVID crisis in all three models (see Appendix 1 in the Supplementary Data section for the full details of the sensitive analysis) (mortality: adjusted OR: 0.94, 95% CI: 0.41–2.13; hospitalisation: adjusted OR: 0.59, 95% CI: 0.21–1.65).

## Discussion

The results of this article are based on two parallel longitudinal studies with the same design, methodology, experimental field (the French West Indies) and evaluation team. Older adults in foster families and nursing homes had comparable socio-demographic and clinical profiles, particularly regarding comorbidities and cognitive status, except for hypertension (more prevalent in nursing homes) and dependency (more pronounced in foster families). Hypertension was self-reported and collected from both older adults and professional caregivers. It is possible some family caregivers interviewed were unaware of untreated hypertension in older adults they cared for, particularly since no differences were observed in the prevalence of cardiac comorbidities. After 1 year of follow-up, the incidences of mortality, hospitalisations and emergency department visits were similar between the two models. Therefore, for older adults who chose foster families over nursing homes, we observed no difference in major health events. Our findings align with those of a small, randomised study (*n* = 112) conducted in 1987 by Oktay and Volland [15], which reported comparable 1-year mortality rates between older adults placed in nursing homes (32%) and those in foster homes (28.8%). Additionally, the very low rate of transition from foster families to nursing homes (*n* = 2), despite the natural deterioration in the health of older adults, suggests that professional foster families can serve as an alternative and not merely a transitional model to nursing homes.

Regarding the evolution of geriatric syndromes, we observed no difference in cognitive decline between the two models. However, we noted a modest but greater deterioration in the dependence score in foster families compared to nursing homes. This point warrants further exploration, possibly linked to the home environment or reduced physical activity. Nevertheless, no difference was observed in the evolution of the percentage of residents in total dependence at one year. A higher level of HrQoL, recognised as a valuable dimension in assessing the benefits of health interventions [24], was observed in foster families. This can be attributed to a more familial context where older adults form strong bonds with their caregivers and other residents [25]. Indeed, notions of mutual support, freedom, a positive outlook on life in foster care, and the feeling of experiencing a pleasant phase of life were reported in one of our previous study [17].

Therefore, foster families for older adults could address the primary concern of older individuals, which is to have a more ‘home-like’ environment than nursing homes. Additionally, they could address the main concern of relatives or medical staff, which is to ensure that major adverse health events are not more frequent in foster families.

The advantages of the professional foster family model, as implemented in the French West Indies, are numerous. Institutional support in terms of initial training, monitoring and financing, along with daily paramedical support, ensures effective and secure care and reassurance for their families. Additionally, these foster families are often geographically close to the families of the older adults, facilitating frequent visits. From an economic perspective, foster families cost half as much as nursing homes in the French West Indies [18] (i.e. 3196 euros), while hospitalisation rates were similar. However, a detailed analysis of direct and indirect costs is necessary, including the cost of paramedical staff in foster families (daily or twice-daily nurse visits, consultations with general practitioners and specialists).

The model does have weaknesses that need to be addressed. More extensive initial and ongoing training on geriatric syndromes is necessary, especially given the profile of the older adults, many of whom are in a state of severe dependence after a few months or years in foster families. Training on malnutrition, neurocognitive impairment and behavioural symptoms of dementia, dependence, and end-of-life care is specifically requested by foster families caregivers. Moreover, foster families in the French West Indies do not have currently a respite system. Finally, foster families may not be suitable for certain types of care or pathologies, such as older adults with active cancer or severe renal failure. In such cases, nursing homes or specialised facilities remain necessary.

**Figure 2:**
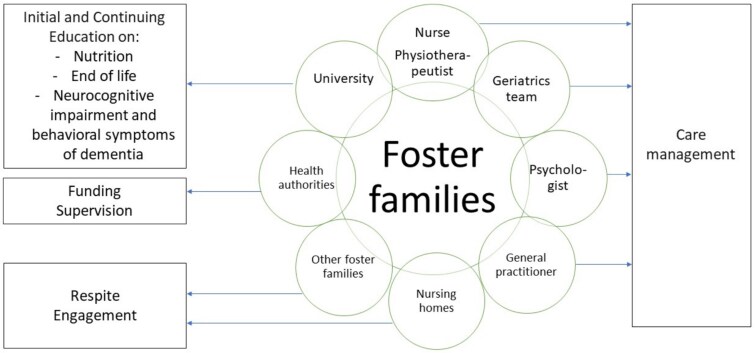
Proposed structure and stakeholders of the foster family care model for countries considering its implementation.

The highly encouraging results of the KASA project suggest that an organised model of foster families could be one of the solutions to address the global ageing of the population. In high-income countries, foster families for dependent older adults could be an alternative to nursing homes, allowing for a diversification of accommodation options when home-based care [4–6] is no longer possible or desirable. In low- and middle-income countries, this may address a need in contexts where nursing homes have not been developed or are not intended to be developed. This model could be built and adapted according to the social, cultural, and economic characteristics of each country, with coordinated organisation of services and partners (Figure 2). For instance, universities may contribute to both the initial and continuing education of caregivers. Financial support for foster families could be provided by public authorities. Respite care may be offered through nursing homes or alternative foster families. The involvement of healthcare professionals could be organised by assigning a dedicated nurse and physical therapist to each family, complemented by targeted interventions from geriatric teams, psychological support and ongoing medical follow-up in collaboration with a general practitioner.

Our study has several limitations. First, to conduct a strict comparison of effectiveness between the two models, a randomised study of the two types of accommodations would have been necessary, which is difficult to accept and implement for older adults and their families. Moreover, our goal was not to oppose these two models, which can be complementary and meet distinct needs and expectations based on the clinical, social and familial profile of the older adults [26]. The second limitation of our study is the follow-up period, which occurred during the peaks of the COVID-19 pandemic [27, 28]. Although we did not observe a difference in mortality rates in nursing homes based on the study period, it is possible that the quality of life was more impacted in nursing homes [29]. Residents in nursing homes benefit from more comprehensive medical monitoring, which may partly explain the observed differences in the detection and management of certain conditions, including hypertension. Finally, the sample size of foster family residents was limited despite a high participation rate. The hospitalisation rate, in particular, was notably low, which can be explained by high rate of paramedical staff, notably during night in French Caribbean nursing homes. Larger-scale studies including older adults from the moment they enter foster families and nursing homes would be necessary.

## Conclusion

Our study suggests that the incidence of mortality and hospitalisations was similar for older adults residing in nursing homes and professional foster families. In a constrained economic context, with the costs of land, building construction, training, recruiting and retaining staff, and a distant time horizon for implementing a nursing home, organised models of professional foster families adapted to the cultures, clinical profiles of the older adults and the socio-economic context of each country deserve to be experimented and supervised by authorities.

## Supplementary Material

aa-25-1311-File002_afaf304
