# Supplementary material for: Comparison of mortality and hospitalisation rates amongst older adults residing in professional foster families versus nursing homes: two parallel observational studies
Source: Age Ageing. 2025 Oct 18;54(10):afaf304. doi: 10.1093/ageing/afaf304 (PMC12535258; doi:10.1093/ageing/afaf304)
Supplement: aa-25-1311-File002_afaf304 [file aa-25-1311-file002_afaf304.docx]

**Comparison of mortality and hospitalization rates among older adults residing in professional foster families versus nursing homes. Two parallel observational studies.**

**Appendix 1: Comparison of deaths and hospitalizations between older adults living in nursing homes included during the COVID crisis and after the COVID crisis. Logistic regression model.**

| **Clinical outcomes** | **Model 1 (Non adjusted)**  **(n=324)** | **Model 2 (adjusted on age, gender)**  **(n=324)** | **Model 3 (adjusted on age, gender, ADL and MMSE score)**  **(n=256)** |
| --- | --- | --- | --- |
|  | OR (CI 95%) | OR (CI 95%) | OR (CI 95%) |
| **Death** |  |  |  |
| Residents included during the COVID crisis | Ref | Ref | Ref |
| Residents included after the COVID crisis | 0.52 (0.27-1.01) | 0.46 (0.23-0.91) | 0.94 (0.41-2.13) |
| **Hospitalizations** |  |  |  |
| Residents included during the COVID crisis | Ref | Ref | Ref |
| Residents included after the COVID crisis | 0.87 (0.36-2.12) | 0.82 (0.33-2.03) | 0.59 (0.21-1.65) |
